# Highly specific and efficient primers for in-house multiplex PCR detection of *Chlamydia trachomatis*, *Neisseria gonorrhoeae*, *Mycoplasma hominis* and *Ureaplasma urealyticum*

**DOI:** 10.1186/1756-0500-7-433

**Published:** 2014-07-06

**Authors:** Ma Guadalupe Aguilera-Arreola, Ana María González-Cardel, Alfonso Méndez Tenorio, Everardo Curiel-Quesada, Graciela Castro-Escarpulli

**Affiliations:** 1 Medical bacteriology, Department of Microbiology, Escuela Nacional de Ciencias Biológicas of Instituto Politécnico Nacional (ENCB-IPN), Esq. Prol. Carpio y Plan de Ayala s/n Col. Santo Tomás, Del. Miguel Hidalgo CP 11340, Mexico DF; 2 Laboratory of Biotechnology and Genomic Bioinformatics, Department of Biochemistry, Escuela Nacional de Ciencias Biológicas of Instituto Politécnico Nacional (ENCB-IPN), Esq. Prol. Carpio y Plan de Ayala s/n Col. Santo Tomás, Del. Miguel Hidalgo CP 11340, Mexico DF; 3 Genetic engineering, Department of Biochemistry, Escuela Nacional de Ciencias Biológicas of Instituto Politécnico Nacional (ENCB-IPN), Esq. Prol. Carpio y Plan de Ayala s/n Col. Santo Tomás, Del. Miguel Hidalgo CP 11340, Mexico DF; 4 Special test laboratory, CMN “20 de Noviembre” ISSSTE, Av. Félix Cuevas #540, Col. Del Valle, CP 03229 Del. Benito Juárez, México DF

**Keywords:** Multiplex PCR, Cervicitis, Detection, *16S rDNA* genes

## Abstract

**Background:**

Although sophisticated methodologies are available, the use of endpoint polymerase chain reaction (PCR) to detect *16S rDNA* genes remains a good approach for estimating the incidence and prevalence of specific infections and for monitoring infections. Considering the importance of the early diagnosis of sexually transmitted infections (STIs), the development of a sensitive and affordable method for identifying pathogens in clinical samples is needed. Highly specific and efficient primers for a multiplex polymerase chain reaction (m-PCR) system were designed *in silico* to detect the *16S rDNA* genes of four bacteria that cause genital infections, and the PCR method was developed.

**Methods:**

The Genosensor Probe Designer (GPD) (version 1.0a) software was initially used to design highly specific and efficient primers for in-house m-PCR. Single-locus PCR reactions were performed and standardised, and then primers for each locus in turn were added individually in subsequent amplifications until m-PCR was achieved. Amplicons of the expected size were obtained from each of the four bacterial gene fragments. Finally, the analytical specificity and limits of detection were tested.

**Results:**

Because they did not amplify any product from non-STI tested species, the primers were specific. The detection limits for the *Chlamydia trachomatis*, *Neisseria gonorrhoeae*, *Mycoplasma hominis* and *Ureaplasma urealyticum* primer sets were 5.12 × 10^5^, 3.9 × 10^3^, 61.19 × 10^6^ and 6.37 × 10^5^ copies of a DNA template, respectively.

**Conclusions:**

The methodology designed and standardised here could be applied satisfactorily for the simultaneous or individual detection of *Chlamydia trachomatis*, *Neisseria gonorrhoeae*, *Mycoplasma hominis* and *Ureaplasma urealyticum*. This method is at least as efficient as other previously described methods; however, this method is more affordable for low-income countries.

## Background

Cervicitis is an inflammatory condition of the cervix. This condition is typically the consequence of an infection with a sexually acquired pathogen, most commonly *Chlamydia trachomatis* and/or *Neisseria gonorrhoeae*. Cervicitis is a frequent cause of dyspareunia, abnormal mucopurulent discharge, friability and menstrual cycle disorders [1,2]. However, asymptomatic rates are as high as 30-45% in some populations [2]. The major concerns regarding undiagnosed or incorrectly diagnosed asymptomatic patients are reproductive sequelae or complications in the upper genital tract [2]. Until an efficacious vaccine is developed, screening and treatment programs appear to be the best method for disease prevention of the “silent epidemic” of *C. trachomatis *[3]. Gonorrhoea, which is caused by *N. gonorrhoeae*, is a common sexually transmitted infection (STI) that is associated with high morbidity and significant socioeconomic consequences. *N. gonorrhoeae* has repeatedly demonstrated the ability to develop resistance to all antimicrobial drugs introduced as first-line therapy [4]. Surveillance of both of these infectious diseases is critical for an accurate estimation of disease incidence and for appropriate prevention and treatment [5].

*Mycoplasma* and *Ureaplasma* species are commonly found in the female genital tract. Although there is an on-going debate, evidence that these microbes cause lower genital tract diseases, including cervicitis, in women is accumulating. Since *Ureaplasma urealyticum* has been divided into *U. urealyticum* and *U. parvum*, the roles of these species in causing urethritis and cervicitis have become even less clear [6]. The accurate diagnosis of *Ureaplasma* spp. and *Mycoplasma hominis* in cervical samples is important because these microorganisms could be pathogenic and could be associated with adverse pregnancy outcomes, postpartum sepsis, neonatal systemic inflammatory response syndrome and bronchopulmonary dysplasia [1].

Diagnosis by nucleic acid amplification tests (NAATs) is available for routine clinical use and is the preferred and recommended method for detecting *C. trachomatis* and *N. gonorrhoeae* infections. The higher sensitivity of these tests is their main advantage over tissue culture and other non-culture-based tests (e.g., enzyme immunoassay, DNA probes) [7]. At present, fully and semi-automated NAATs are commercially available. One disadvantage of this method is the higher cost [7]. However, in high-income countries, the increased test sensitivity might make NAATs more cost-effective than other tests because the use of NAATs might decrease the prevalence of these genital infections [8]. However, in low-income countries, the start-up cost of introducing commercial NAATs might be prohibitive. Therefore, there is an urgent need for improved and cost-effective diagnostic tests that would reduce the burden of STIs and allow for the creation of mass screening programs to avoid the spread of these diseases.

Data regarding the relative frequencies of these microorganisms in the Mexican population are minimal [9-11] Moreover, infections caused by *Chlamydia*, *Mycoplasma* and *Ureaplasma* species have not been adequately studied in developing countries, and their prevalence is under-researched. Considering the effect that infection or colonisation by *C. trachomatis, N. gonorrhoeae, M. hominis* and *Ureaplasma* spp. might have on the female reproductive tract and the high number of unrecognised infected individuals, who might serve as reservoirs for spreading infections to men and other women, there is an urgent need for early and accurate diagnosis [1].

In the present study, highly specific and efficient primers for the in-house multiplex polymerase chain reaction (m-PCR) detection of *C. trachomatis*, *N. gonorrhoeae*, *M. hominis* and *U. urealyticum* were bioinformatically designed based on specific and conserved genetic sequences; these primers and the method were then standardised in the laboratory to rapidly, inexpensively and effectively detect the four bacteria commonly associated with cervicitis in a single reaction (quadruple/qx-PCR).

## Methods

### Biological materials

*C. trachomatis* ATCC® VR-878D™ DNA, *M. hominis* ATCC® 23114D™ DNA and DNA from cultures of *U. urealyticum* ATCC® 27618™ and *N. gonorrhoeae* ATCC® 53420™ were used for the in vitro standardisation assays. *U. urealyticum* DNA was obtained using the NucliSens® system (bioMérieux, Marcy l’Etoile, France), and *N. gonorrhoeae* DNA was obtained using the InstaGene™ matrix (Bio-Rad Laboratories, Inc., Hercules, CA, US) according to the manufacturer’s instructions. *Streptococcus agalactiae* ENCB 580 M16 and *Staphylococcus saprophyticus* ENCB, *Lactobacillus acidophilus* ATCC® 9224™ and *Gardnerella vaginalis* ATCC® 14018™ strains were grown overnight on blood-based agar (Bioxon, Becton, Dickinson and Company, México), MRS agar and Casman agar plates, respectively, at 37°C. *L. acidophilus* ATCC® 9224™ and *G. vaginalis* ATCC® 14018™ were incubated in an atmosphere of 5% CO_2_. Two hundred thirty-seven cervicovaginal exudates were collected with approval from the ethics committee of the National Medical Centre November 20 (Centro Médico Nacional 20 de Noviembre) approval number 02/09 at the Institute of Security and Social Services for State Workers (Instituto de Seguridad y Servicios Sociales de los Trabajadores del Estado, ISSSTE), Mexico City. Written informed consent was obtained from all of the study participants. All of the clinical specimens were analysed by routine microbiological methods (data not shown), and the clinical specimens that were negative for the presence of genital infections were used for the analytical specificity determinations (see below).

### Primer design

The Genosensor Probe Designer (GPD) (version 1.0a) software [12] was initially used to evaluate critical primer parameters, including the melting temperature (Tm), difference in melting temperatures for primer pairs (∆Tm), GC content (GC%), self-complementarity, repetitive sequences and Gibbs free energy. Primers with acceptable physicochemical parameters were selected and aligned with the sequence from the National Center for Biotechnology Information (NCBI) database using the Basic Local Alignment Search Tool (BLAST) to test for possible nonspecific interactions. Some primers had nonspecific matches with genomic sequences of other common inhabitants of the vaginal cavity. BioEdit sequence alignment editor (version 7.0) software was used to align the primers with gene sequences to identify the most variable regions (highest entropic regions) and to find new regions for designing specific primers. GPD was used again to examine the new primer sequences (Tm, ∆Tm, GC%, self-complementarity, repetitive sequences and Gibbs free energy). WinOligo, a specialised component of the GPD software, was used for calculating bimolecular secondary structures, including primer-primer interactions (hairpins, homodimers or heterodimers), that might affect the yield or the success of amplification reactions.

The designed primer set is a combination of four primer pairs that amplify species specific *16S rDNA* target regions of C*. trachomatis* (402 bp), *N. gonorrhoeae* (694 bp), *M. hominis* (604 bp) and *U. urealyticum* (898 bp) from genomic DNA. The amplicon sizes were selected during the primer design to allow for co-amplification and appropriate separation on a 1.8% agarose gel by electrophoresis to facilitate the unambiguous identification of the PCR products by size.

### PCR method

Single-locus PCR reactions were performed and standardised. PCR amplification was performed in a final volume of 50 μL containing 100 ng of DNA template, 0.4 mM deoxynucleotide triphosphate (dNTP) mix, 1x PCR buffer (Invitrogen™, Carlsbad, CA), 2 mM MgCl_2_, 0.1 μM primers, 2.5 units of *Taq* polymerase (Invitrogen™, Carlsbad, CA). To prevent PCR inhibition, 0.4% bovine serum albumin (BSA) was added. The following sequences of the designed primers are under patent application (MX/A/2011/011064): 16SCt-S-5’-CGA GTC GGC ATC TAA TAC TAT-3’, 16SCt-AS-5’-AAA ACG ACA TTT CTG CCG C-3’, 16SUu-S-5’-TAC CCT TAA GTT GGG GAT AA-3’, 16SUu-AS-ACT ATA TTT CTA TAG CGT CGC AA-3’, 16SNg-S-5’-GCC TCG CGG CTT GGC TA-3’, 16SNg-AS-GGC GCA GAC GGT TAC TTA AGC AGG A-3’, 16SMh-S-5’-ACC CAT TGG AAA CAA TGG CTA ATG CCG GAT ACG-3’ and 16SMh-AS-5’-ATA GAC CCA GTA AGC TGC CTT CGC CT-3’. All of the reactions were performed in a T-Gradient Thermoblock PCR System (Biometra, Goettingen, Germany). The cycling conditions to amplify all of the individual loci (s-PCR) consisted of an initial denaturation at 94°C for 5 min, 30 cycles of melting at 94°C for 1 min, annealing at 58°C for 30 s, and elongation at 72°C for 1 min and a final extension at 72°C for 5 min. Primers for each locus were added in sequential amplifications until qx-PCR was achieved; each locus was added individually because combining the primers in various mixtures and amplifying many loci simultaneously typically required alteration/optimisation of the reaction parameters [13]. A minimum of 2 replicates were performed for each reaction. To verify the presence of the amplification products, 10 μL of each PCR was separated by electrophoresis on a 1.8% agarose gel. A 100-bp DNA molecular marker was included in some of the electrophoresis runs (Invitrogen™, Carlsbad, CA). The remaining 40 μL of the amplification reaction was frozen for later use. The products of s- and qx-PCR reactions of each target gene were authenticated via DNA sequencing on an ABI-PRISM^TM^ 310 (Applied Biosystems, Foster City, CA) using the manufacturer’s recommendations.

### Generation of PCR amplification controls

After all of the amplicons were obtained, they were cloned into a pJET1.2/blunt vector using the CloneJET™ PCR Cloning Kit (Fermentas Life Sciences, Dharmacon, Inc.), and *Escherichia coli* DH5α competent cells were transformed to produce sufficient positive amplification controls. Plasmid DNA from characterised transformants was purified using CHROMA SPIN™ columns (Clontech, Mountain View, CA) and was used as a positive control in all of the PCR assays. Equimolar concentrations of these amplicons were mixed and used as molecular weight markers.

### In vitro standardisation

The analytical specificity was tested in two steps. In the first step, suspensions of *S. agalactiae*, *L. acidophilus*, *G. vaginalis* and *S. saprophyticus* adjusted to 0.5 McFarland Nephelometer standard, which is equal to 1.5 × 10^8^ colony-forming units (CFU)/mL, were prepared. Genomic DNA was extracted using the High Pure PCR Template Preparation Kit (Roche, Mannheim, Germany), and s- and qx-PCR were performed. In the second step, adjusted suspensions were added to negative cervical samples, which were transported and maintained in 2-sucrose-phosphate (2SP) medium. Metagenomic DNA was extracted (High Pure PCR Template Preparation Kit), and s- and qx-PCR were performed to determine the analytical specificity of a clinical sample. In addition, the absence of PCR inhibitors in the negative amplification reactions was validated by re-amplification with the addition of plasmid DNA from the four characterised transformants as positive controls. Two replicates were performed for each reaction, and the results were consistent.

Genomic DNA from *C. trachomatis, N. gonorrhoeae, M. hominis* and *U. urealyticum* was diluted in 10-fold serial dilutions, and s- and qx-PCR were performed to determine the limits of detection. Two replicates were performed for each reaction, and the results were consistent.

## Results

### Primer design

Conserved genes were selected as the target genes, and suitable primers were designed to detect four cervicitis-related bacteria. The following general requirements for design were considered: the primers have similar Tm values; the primers would not form hairpins, homodimers or heterodimers or have repetitive sequences; and the target fragment gap should exceed 30 bp to meet the resolution requirements for agarose gel electrophoresis. Using GPD and its WinOligo tool, highly specific and efficient primers were generated. These primers were designed with similar physical characteristics to allow for simultaneous amplification under the same conditions in single or multiplexed reactions. The lengths of the primers ranged from 17 to 33 bp, and their melting temperatures were between 68 and 77°C. These characteristics are important because longer primers allowed the reaction to be performed at a higher annealing temperature and yielded fewer nonspecific products. No undesirable primer-primer interactions or repetitive sequences were detected. The expected amplicon sizes ranged from 402 to 989 bp. A key step in creating the primers was finding the more variable (highly entropic) regions inside of each of the *16S rDNA* sequences and using these areas as species-specific regions.

### *In vitro* standardisation

**Figure 1:**
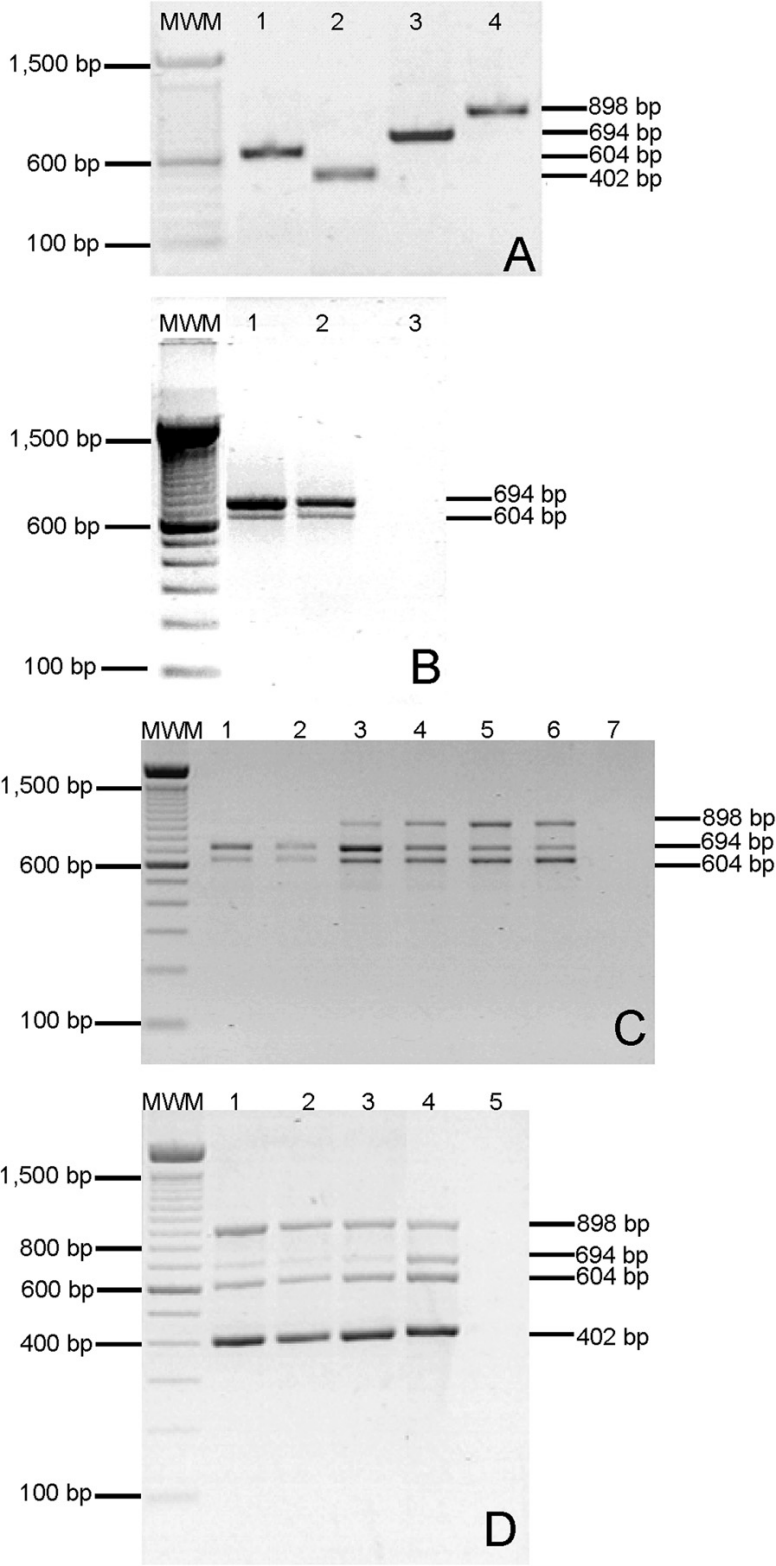
**Representative images of the standardisation process for the detection of *C. trachomatis, N. gonorrhoeae, M. hominis *and *U. urealyticum *by *16S rDNA *gene amplification.** Lanes MWM: 100-bp marker (Invitrogen™, Carlsbad, CA). The sizes (bp) are indicated on the left. The PCR products were electrophoresed on a 1.8% (wt/vol) agarose gel, stained with ethidium bromide and photographed under UV light. **A**. Lanes 1-4: Single PCR detection of *M. hominis*, *C. trachomatis, N. gonorrhoeae* and *U. urealyticum*. **B**. Lanes 1-2: Duplex PCR for the simultaneous detection of *M. hominis* and *N. gonorrhoeae*; lane 3: negative control (no template). **C**. Lanes 1-6: Triplex PCR for the simultaneous detection of *M. hominis*, *N. gonorrhoeae* and *U. urealyticum*; lane 7: negative control. **D**. Lanes 1-4: Quadruplex PCR for the simultaneous detection of *M. hominis*, *N. gonorrhoeae*, *U. urealyticum* and *C. trachomatis*; lane 5: negative control. Panels **B**, **C** and **D** also show the effects of the target DNA concentration on the multiplex amplifications; the best yield is shown in lanes 2, 6 and 4 using 100 ng of DNA template.

The optimisation process was first performed using each pair of primers in a separate amplification reaction (single), and annealing temperatures ranging from 56°C to 70°C were tested. The optimal temperature was 58°C under standard concentrations of the PCR components. The concentrations of the primers, the PCR co-adjuvant (BSA) and the target DNA were adjusted. Amplicons of the expected size were obtained from each of the four bacterial gene fragments. The banding patterns from the gel analysis are shown in Figure 1A. Multiplexed reactions were performed individually in an empirically chosen order until quadruplex amplification was achieved. In this phase, no parameters or cycling conditions were re-adjusted. The target DNA concentrations were adjusted to enhance amplification. Panels **B**, **C** and **D** show the effects of the target DNA concentration on the multiplex amplifications; the best yield is shown in lanes 2, 6 and 4, respectively, using 100 ng of target DNA.

To evaluate the specificity of the multiplex assay, a panel of 5 well-characterised strains were employed in the test (*S. agalactiae*, *S. saprophyticus*, *L. acidophilus, G. vaginalis* and *N. gonorrhoeae*). The amplification results using the multiplex assay are shown in Table 1. The primers yielded a PCR product only when *N. gonorrhoeae* was present, as was expected. The primers did not amplify any product from a non-STI species (Table 1).

**Table 1:** Evaluation of PCR primer analytical specificity

**Inoculated microorganism**	**Analytical specificity results**			
	**Addition of:**		**Amplification result in:**	
	**2SP**	**Negative clinical sample**^**a**^	**Single PCR**	**Quadruplex PCR**
*N. gonorrhoeae*	Yes	Yes	+	+
*G. vaginalis*	Yes	Yes	-	-
*L. acidophilus*	Yes	Yes	-	-
None	Yes	Yes	-	-
Mix^b^	Yes	Yes	-	-
*S. agalactiae*	Yes	No	-	-
*N. gonorrhoeae*	Yes	No	+	+
*S. saprophyticus*	Yes	No	-	-
None	Yes	No	-	-
Mix^b^	Yes	No	-	-

+ = presence of amplicon.

- = no amplicon was visualised on agarose gel.

^a^The negativity of the samples was demonstrated by the detection of cervicovaginal pathogens using culture, Mycoplasma IST 2 – API galleries (bioMérieux, France) and/or PCR (data not shown).

^b^The mixes include *G. vaginalis, L. acidophilus, S. agalactiae* and *S. saprophyticus*.

2SP: 2-sucrose-phosphate transport medium.

The detection limit for each primer set in the multiplex format was also determined. To determine the limits, 10-fold serial dilutions of known amounts of *C. trachomatis, N. gonorrhoeae, M. hominis* and *U. urealyticum* DNA were tested in triplicate using the multiplex format. The detection limits for the *C. trachomatis*, *N. gonorrhoeae*, *M. hominis* and *U. urealyticum* primer sets were 5.12 × 10^5^, 3.9 × 10^3^, 61.19 × 10^6^ and 6.37 × 10^5^ copies of a DNA template, respectively.

## Discussion

The ribosomal *16S* gene is a suitable molecular target for bacterial classification because this gene is universal among bacteria and is conserved, although it has sufficient variation (nine hypervariable regions) to discriminate between the majority of taxa (species-specific sequences). One PCR primer pair could target the *16S rRNA* gene from a wide range of bacterial species [14]. Taking advantage of these characteristics, a bioinformatics study was performed to develop highly specific and efficient primers for the in-house m-PCR detection of *C. trachomatis*, *N. gonorrhoeae*, *M. hominis* and *U. urealyticum*.

The m-PCR technique could be used as a differential method to determine whether an STI or cervicitis is caused by one of the previously mentioned bacteria, and this technique could be a valuable tool for reducing the time required to make a diagnosis and initiate treatment. Because asymptomatic infections have been described in the literature, simple, practical and inexpensive methods that improve the diagnoses of these bacterial infections are necessary. These methods could be used to monitor the prevalence of these bacteria through free routine tests, which could be implemented in a surveillance project to prevent further transmission of the disease [15,16].

Traditionally, testing for *C. trachomatis* and *N. gonorrhoeae* is performed on cervical swabs that are sent for culture, phenotypic or immunological detection [7]. These tests are often not performed immediately, and it could take more than two days for the results to become available. Although several commercial tests (NAATs) are an option for *C. trachomatis* and *N. gonorrhoeae* detection, these tests are usually more expensive and are not readily available in some labs because the appropriate infrastructure is required [6]. These issues are major deterrents to using these kits to implement the large-scale screening programmes that should be conducted in low-income countries. Additionally, because of the high risk of sequelae from untreated *C. trachomatis* or *N. gonorrhoeae*, physicians have been told to practice symptom management and empirically treat patients who are suspected of having active *C. trachomatis* or *N. gonorrhoeae* infection. Otherwise, patients might not return for their lab results and would not wait 2 or 3 days to begin treatment. The availability of a test such as the one described herein (in-house m-PCR), which provides results within 3 or 4 hours and allows for immediate treatment, is relevant because this test reduces the risk of potential onward transmission and sequelae. Because symptom management misses patients with mixed venereal diseases and asymptomatic patients, the NAATs as a qx-PCR approach are highly needed [6,17,18].

Although the roles of *M. hominis* and *U. urealyticum* as aetiological agents of cervicitis have yet to be clarified, their presence in the female reproductive tract has been correlated with adverse pregnancy outcomes and adverse consequences to newborn babies. Therefore, it is important to determine the relative frequencies of these agents in different populations [1] and their co-prevalence with clinical entities such as vaginitis, bacterial vaginosis and cervicitis.

M-PCR is a rapid tool that allows for the simultaneous amplification of more than one sequence of target DNA in a single reaction, saving time and reagents [13]. To the best of our knowledge, this study is the first report of in-house qx-PCR for this specific combination of etiological agents. In addition, these primers could be used to amplify one to all four of the different targets, making this m-PCR a flexible technique to use with several types of clinical samples. For example, this method could be used for single or simultaneous detection in the diagnosis of the causes of trachoma (*C. trachomatis*), atypical pneumonia (*C. trachomatis*, *U. urealyticum*) or non-gonococcal urethritis (*C. trachomatis*, *M. hominis* and *U. urealyticum*).

The analytical specificity of PCR depends on the primers used, and the analytical sensitivity depends on the presence of inhibitory substances in the specimens [18]. The specificity of the primer combinations was demonstrated by the negative PCR results obtained with all of the non-STI reference strains, and the addition of BSA in the PCR reaction was a good strategy to minimise the effect of inhibitory substances in cervical specimens because there was no amplification in the PCR reactions without BSA (data not shown). However, the clinical specificity and clinical sensitivity of the designed PCR technique should be evaluated in a statistically significant sample.

Recently, several researchers used in-house or commercial PCR assays to identify STI bacteria by detecting the *16S rDNA* gene or other genes in s-PCR or m-PCR. Early work by Patel et al. [19] reported the prevalence of *Chlamydia* infection among women in India, which was analysed via in-house s-PCR designed to amplify the *gyrA* gene of *C. trachomatis*. Corbeto et al. [20] determined the prevalence of *C. trachomatis* infection using a commercial real-time PCR method in Catalonia, Spain. In contrast to those studies, the qx-PCR technique described in this study requires a single reaction to detect four bacterial genes related to cervicitis. Other authors have reported a simple design that involved standardising five s-PCR techniques to specifically investigate the relative frequencies of *C. trachomatis* and genital mycoplasmas in Brazil. These s-PCR techniques are capable of amplifying the target sequence on a cryptic plasmid of *C. trachomatis*, the *cppB* gene of *N. gonorrhoeae*, the major adhesion protein gene of *Mycoplasma genitalium* and the urease gene of *U. urealyticum* in five different reactions [1], whereas the qx-PCR technique described here could be used for the simultaneous detection of these species.

Perhaps the most complex m-PCR technique was described by McKechnie et al. [21]. These authors reported the simultaneous identification of 14 genital microorganisms, including *C. trachomatis*, *N. gonorrhoeae*, *M. hominis* and *U. urealyticum*, in the urine of human males using an m-PCR-based reverse line blot assay (m-PCR/RLB). The set of targets examined by these authors is better suited to the detection of important aetiological agents of male urethritis [21]. The authors concluded that their m-PCR/RLB technique could be used for routine diagnosis and epidemiological studies because it is more practical and less expensive than microarray technology. The qx-PCR technique described here could represent a more widely applicable method for analysing samples because it is cheaper, easier to perform and comparable in sensitivity to m-PCR/RLB.

De Haro et al. [9] used an s-PCR (t-PCR) technique to amplify the *omp*A gene of *C. trachomatis* in 152 endocervical samples from infertile women, and Hernández-Martínez et al. [10] use two s-PCR to detect *C. trachomatis* or *N. gonorrhoeae* and phenotypic kits to identify genital mycoplasmas in 105 endocervical samples from the general population. Although the De Haro and Hernández-Martínez studies were similar in design, the latter study characterised a wider range of bacteria. Both studies attempted to determine the relative frequencies of various genital infectious agents and to determine which species are the most plentiful in different populations. In the context of these previous studies, the qx-PCR technique described here could be an improved new method. *C. trachomatis*, *M. hominis* and *U. urealyticum* are widespread in females who were not considered at-risk, and *C. trachomatis* infections were diagnosed in asymptomatic patients [10]. These results increase our interest in generating a rapid, inexpensive and practical new methodology that is applicable to a larger number of samples in a short time and is not dependent on sophisticated technology.

The qx-PCR system reported in this study reduces analysis time and promises to be a tool that provides more accurate risk assessments and could be used for monitoring the pathogenic strains of the most frequently identified cervicitis agents. The qx-PCR method exhibited acceptable specificity and sensitivity even when the clinical samples were suspended in a transport medium such as 2SP. However, these results should be confirmed with a larger set of samples in a future study to evaluate the clinical specificity and sensitivity.

## Conclusions

We developed and evaluated a qx-PCR assay for the rapid detection of cervicitis-related bacteria. Although sophisticated methodologies have been designed, using endpoint PCR to detect *16S rDNA* genes remains a good approach for estimating the incidence and prevalence of several bacteria and for monitoring infections. The qx-PCR technique would be valuable as a simple tool to screen for the presence of these bacteria because the specific combination of genes employed facilitates the identification of these bacteria in the various types of clinical samples that are commonly analysed in clinical, research and reference laboratories.

## Competing interests

The authors declare that they have no competing interests.

## Authors’ contributions

AGC performed the molecular genetic studies and bioinformatics analyses. AMT provided the software and supervised the bioinformatics design and results. GCE participated in the data analysis and assisted with the draft of the manuscript. ECQ provided advice and performed the cloning and transformation assays. MGAA conceived the study, participated in the study design and coordination, assessed the data and drafted the manuscript. All of the authors read and approved the final manuscript.
